# The Actual Demand for the Elimination of Architectural Barriers among Senior Citizens in Poland

**DOI:** 10.3390/ijerph16142601

**Published:** 2019-07-22

**Authors:** Izabela Kurtyka-Marcak, Maria Hełdak, Katarzyna Przybyła

**Affiliations:** 1 Institute of Economics Sciences, Wroclaw University of Environmental and Life Sciences, 50-375 Wrocław, Poland; 2 Department of Spatial Economy, Wroclaw University of Environmental and Life Sciences, 50-375 Wrocław, Poland

**Keywords:** architectural barriers, mobility problems of senior citizens, elimination of architectural barriers

## Abstract

The purpose of the study is to determine the actual demand for the elimination of architectural barriers among senior citizens in their place of residence and also in its immediate environment in Poland. The research covered a group of people in the post-productive age, living in the Lower Silesia voivodship in Poland. Different research methods were used in the study, primarily including the public opinion survey based on a questionnaire as well as statistical analyses. The cross-tabulation analysis of differences in quality characteristics was performed using Pearson’s chi-square test (χ^2^ test of independence) or Fisher’s exact test, when the expected number was lower than five. As a post hoc analysis, checking the nature of differences between the studied groups, the analyses were carried out using the method by Baesley and Schumacker. For all analyses, the maximum permissible error class I α = 0.05 was adopted, whereas *p* ≤ 0.05 was considered statistically significant. The research revealed that a much larger group of people aged 55 and older suffers from mobility limitations than the ones resulting from disability certificates, thus confirming the assumption that along with the respondents’ age, their mobility limitations intensify, resulting in the need for assistance while moving outside their houses/apartments.

## 1. Introduction

Architectural barriers stand for all obstacles occurring in a building and in its immediate environment, which due to technical or construction solutions or usage conditions make it impossible or difficult for people suffering from mobility limitations, such as, e.g., the elderly, people with disabilities, or people moving with a stroller [1]. In Poland, solving the problems of persons with disabilities, i.e., eliminating architectural barriers, which prevent or impede the disabled freedom of movement remains the task of county authorities (the local government unit in Poland) and is co-funded by the National Fund for Rehabilitation of Disabled People (PFRON). The primary objective of the fund is to create conditions which facilitate full participation of the disabled in professional and social life [2]. The fund, however, supports only people with a disability certificate, therefore not everyone who, in fact, needs such help. The actual scale of the problem remains pure conjecture. Simultaneously, it is worth emphasizing the problem of existing spatial diversity in terms of satisfying social needs determining the population living standard, including persons with disabilities [3,4,5,6].

According to Bartkowski [7], the research conducted among the disabled highlighted the importance of various barrier types in their daily functioning. The research findings, covering persons with disabilities, often indicate that the obstacles causing difficulties in getting around and dealing with every day or official issues are on top of the list of their problems. The research carried out by Frąckiewicz [8] showed that the problem of barriers does intensify with age. In older age groups, the living space of persons with disabilities is increasingly limited to their house/apartment and its surroundings. Although much has been done to improve the situation of the disabled since the aforementioned studies were conducted, the assumption is that still a significantly limited space is available for persons with disabilities, predominantly senior citizens.

This group also covers people whose general health is gradually deteriorating and they frequently suffer from more than one disease. In spite of the intensive economic development, which has taken place in recent decades, including the average income increase in Poland [9,10], senior citizens, in particular those living out of town [11] represent the social group characterised by significantly lower incomes and resources, lower possibilities of satisfying their needs, thus often living on the verge of poverty. In view of structural problems within the public service system, they are not always provided with efficient healthcare [12]. An important issue for this group is to receive assistance in meeting their everyday needs, hence barriers of various types are much more strongly perceived and experienced than in earlier periods of life as they limit mobility and thus the possibility of social participation. Therefore, social integration requires a number of activities in the form of financial support, care and assistance in meeting the needs of everyday life. The policy aimed at satisfying “life quality” needs may be of great importance for this particular group [13,14,15,16].

The analyses carried out by Hełdak et al. [13] on the elimination of architectural and technical barriers in Poland became the reason for undertaking the discussed research. In the course of the research, certain problems were encountered in determining the actual number of people with the need for eliminating architectural and technical barriers. Thus the decision was to carry out a health status analysis covering senior citizens (in post-productive age) to determine their scale of mobility limitations. It was acknowledged that the research should be supported by a survey conducted among the post-productive age population. This would allow specifying the actual demand for construction works, as well as alterations in interior finishing aimed at eliminating mobility limitations. It was established that this part of the society hardly ever applies to be granted the status of persons with disabilities.

The activities focused on eliminating architectural barriers in the place of senior citizens’ residence, including their immediate environment are of particular importance in the context of their life quality and independence improvement. As the conducted research has shown, a built environment allowing physical activity is positively correlated with the independence of senior citizens [17]. The conclusions resulting from this research can therefore measurably affect the everyday functioning of people with reduced mobility.

## 2. Materials and Methods

The presented study aimed at identifying the actual demand for eliminating architectural and technical barriers among the elderly, i.e., people aged 55 or older (in Poland this age is associated with the possibility of early retirement). Early retirement can be applied for by the following groups: Women aged 55 and men aged 60. It, however, depends on documenting the contribution and non-contribution periods as of 1 January 1999. The contribution period for women is at least 20 years and for men it is at least 25 years. These years have to cover the period of 15 years working in special conditions.

The article puts forward the following hypotheses:

**Hypothesis** **1.** *A much larger group of people aged 55 and older has mobility limitations than the ones resulting from disability certificates.*

**Hypothesis** **2.** *Houses/apartments of the elderly (55 years and older) are not adjusted to their mobility needs and require significant financial outlays to eliminate architectural barriers.*

Research stages:

- Subject literature review and formulation of the research goal,
- Preparing a questionnaire (survey),
- Conducting surveys in selected areas of Lower Silesia voivodship,
  - Analysis of the obtained results using a descriptive and statistical methods and identifying the following correlations:
    - Suffered mobility limitations vs. the disability certificate and using funds aimed at the elimination of barriers;
    - Facilities used in moving around the house/apartment vs. the actual demand for the elimination of architectural barriers;
    - Mobility limitations imposing the need to use facilities while moving vs. the respondents’ age;
    - Independence in moving outside the house/apartment vs. the respondents’ age;
    - Need for facilities to move in the closest environment (neighbourhood)–outside the house/apartment vs. the respondents’ age;
    - Demand for public utility services in the immediate environment vs. the respondents’ age and place of residence.
- Verification of the research hypotheses.

**Figure 1:**
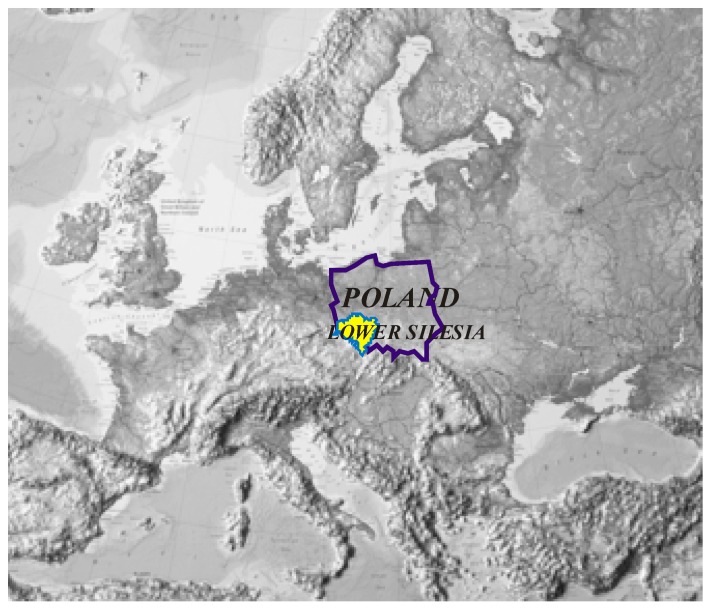
Location of Poland and the Lower Silesia region on the background of Europe. Source: Authors’ compilation.

The research covered the area of Lower Silesia voivodship, situated in south–western Poland. Currently about 2.9 million people (Statistics Poland) reside in Lower Silesia (Figure 1). 

The voivodship is ranked as fifth in Poland in terms of population number. The average age of people living in Lower Silesian cities is 40.9 years of age, whereas in villages it is 37.8 years of age. The total population number is decreasing from year to year, and the number of post-productive age people is continuously growing. The Statistics Poland forecasts that in 2050 the average age of Lower Silesian city residents will increase to 54.6 years of age and in villages to 51.3. Moreover, 400,000 fewer residents than today will be living in the voivodship. The region of Lower Silesia is characterized both by the areas of growth and of depopulation. Unfavorable demographic changes affecting the Sudeten villages were observed throughout the entire post-war period [18,19,20,21,22,23]. A further depopulation of medium-sized towns in the Sudeten and in the Kłodzko Valley region is anticipated. “The region of Lower Silesia is characterized both by the areas of growth and of depopulation. Unfavorable demographic changes affecting the Sudeten villages were observed throughout the entire post-war period [18,19,20,21,22,23]. A further depopulation of medium-sized towns in the Sudeten and in the Kłodzko Valley region is anticipated. The process of population aging is particularly noticeable in the environment of still quite young Wrocław’s society. The statistics, however, show clearly the advancing and raising concerns phenomena in the age structure of the entire region and the country. Poland is still perceived in Europe as a demographically young country, however, since the beginning of the 1990s the average Polish resident has aged by more than seven years [24]. The aging process remains one of the most important challenges and demographic problems of the voivodship [25]. The age median for Lower Silesia in 2017 was 41.3 years of age, 39.7 years for men (in 1990–31.8) and 43 years for women (in 1990–34.3). The fertility rate in Poland in 2017 was 1.45 (in 1990–1.99) in 2018—1.3, whereas for Lower Silesia in 2017 it was 1.36 [26]. Therefore, solutions should be sought to improve “self-sufficiency” (self-reliance) of the aging population”.

The random purposive sampling was used to select the respondents for the survey purposes. The target criterion was the respondent’s age and place of residence in Lower Silesia voivodship. The source materials were collected based on a survey using a questionnaire [27,28]. Random individuals, the total of 214 respondents, within the indicated age group were covered by the study. The authors have not established any guidelines regarding the number of people by gender, age or place of residence. The number of respondents was important. The respondents were asked to answer quite detailed questions about the available facilities improving their mobility comfort in the place of residence (apartment/house/care home). They were expected to determine the currently available facilities and the actual demand for various devices to improve everyday activities of the elderly and the disabled. A specialized terminology, referring to the devices installed inside the buildings to help people with mobility problems was used here. In addition, the respondents were asked about the needs for the respective facilities in the environment of the buildings and the demand for services (taxi rank, grocery store, health care center, church, bus stop, restaurant). The answers were compared against the control questions regarding the age or the character of the location in which the respondent resides. The Annex 1 was changed to Supplementary Material 1 to this article. 

In order to answer the research questions and test the above formulated hypotheses, statistical analyses were carried out using IBM SPSS Statistics package, version 25 (IBM, Armonk, NY, USA). The cross-tabulation analysis of differences in quality characteristics was performed using Pearson’s chi-square test (χ^2^ test of independence) or Fisher’s exact test, when the expected number was lower than five. As post hoc analysis, checking the nature of differences between the studied groups, the analyses were carried out using the method by Baesley and Schumacker [29]. For all analyses, the maximum permissible error class I α = 0.05 was adopted, whereas *p* ≤ 0.05 was considered statistically significant.

## 3. Discussion

### 3.1. Mobility Limitations among the Respondents vs. the Disability Certificate Held and Using Funds for Removing Barriers

The conducted research was primarily focused on the analysis of correlations between the suffered mobility limitations, the disability certificate held and using funds for the elimination of barriers. For this purpose, the analyses using chi-square test of independence or Fisher’s exact test were conducted. Table 1 presents the respondents’ answers regarding the disability certificate, and Table 2 regarding the use of state funds for the elimination of architectural barriers.

The carried out analysis showed that 80% of people holding a disability certificate moved on a wheelchair and their number is significantly higher than the number of wheelchair users who did not have a disability certificate.

In the case of those walking with a crutch, the differences between holding and not holding a disability certificate did not differ significantly.

The persons using a walking frame were the significantly more frequent disability certificate holders than those moving on a wheelchair. Four persons using a walking stick and a person moving by car (Table 2) held a disability certificate.

The conducted analysis showed that among people using wheelchairs, aged 55 and older, 20% of the respondents used state funds for the elimination of architectural and technical barriers and their number is significantly higher than the number of wheelchair users who do not use state funds (7.6%).

In the case of other respondents using a crutch or a walking frame, the differences in the proportions of people using state funds were insignificant.

Among other facilities used to move around; 14 people used a walking stick and one person used a car at a distance up to 1 km—none of them used the aforementioned funds.

### 3.2. The Available Facilities for Moving around An Apartment/House vs. Actual Demand for the Elimination of Architectural Barriers

The next part of the study is focused on determining correlations between the available facilities in moving around an apartment/house and the actual demand for them. For this purpose, a series of analyses were performed using chi-square test of independence or Fisher’s exact test and comparing the proportion of such facilities against those actually needed. The following sections of the article discuss in detail the results regarding different types of facilities.

**Figure 2:**
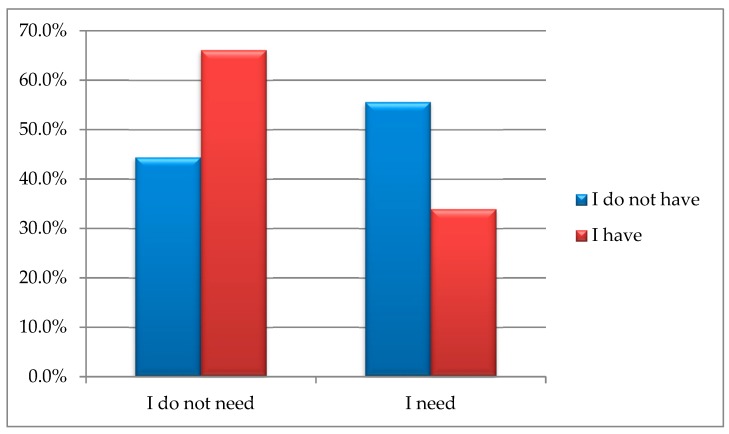
Different available handholds and grippers vs. the actual respondents’ demand for this type of facilities.

In terms of the available facilities against the ones actually needed, such as various handholds or grippers, the analysis conducted using chi-square test of independence showed significant differences between the proportions in the provided answers, i.e., χ^2^(1) = 7.99; *p* = 0.005; φ = −0.19. The findings confirm that the number of people who actually need and do not have such facilities is significantly higher than the number of those who need and have various handholds/grippers. More than half (55.6%; *n* = 85) of the respondents do not have handholds/grippers and claim they should have them available. The next group, 33.9% (*n* = 20) had, and needed, grippers. Figure 2 presents the responses provided by senior citizens.

**Figure 3:**
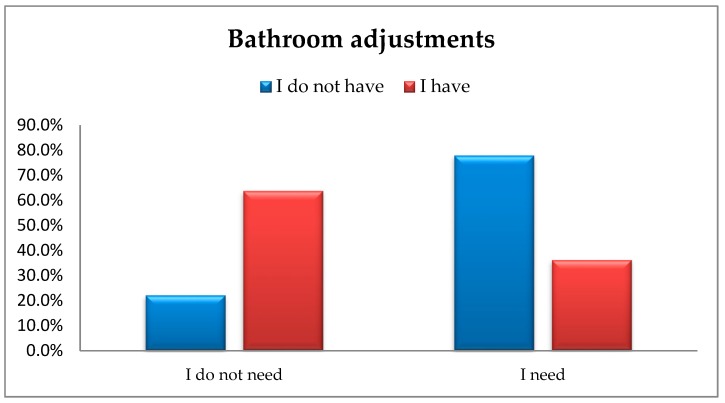
The availability of bathroom adjustments vs. actual respondents’ demand for this type of facility.

Next the adjustment of bathrooms to the needs of older people was analysed. The analysis using chi-square test of independence showed significant differences between the responses provided by the respondents, i.e., χ^2^(1) = 32.92; *p* < 0.001; φ = –0.9 (Figure 3). 

The number of people who need and do not have this type of facility is significantly higher than the number of people who need it and for whom it is actually available in their apartment/house. In total, 36.2% (*n* = 21) of the respondents had and needed an adjusted bathroom, whereas as many as 77.0% (*n* = 120) did not have one, however, they did need an adjusted bathroom.

**Figure 4:**
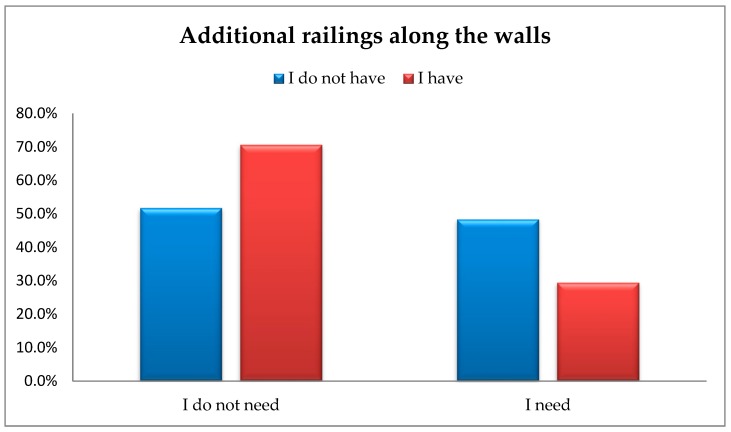
The availability of additional railing along the walls vs. actual respondents’ demand for this type of facility.

The findings reveal that the number of people who do not have, but need additional railings along the walls is significantly lower than the number of people who have this kind of facility available to them. A total of 29.4% (*n* = 10) of the respondents had and needed additional railings along the walls, whereas 48.3% (*n* = 86) did not have, but needed this type of facility. The analysis using a chi-squared test of independence showed significant differences in the proportions of the responses provided by the respondents regarding the availability of additional railings along the walls, i.e., χ^2^(1) = 4.12; *p* < 0.042; φ = –0.14 (Figure 4).

Next, another type of senior citizens’ demand for the facilities in their apartments/houses was analysed. The analysis conducted using a Fisher’s exact test did not show any significant differences in the proportions between the actually available and needed facilities, such as:

- Slip resistant flooring,
- Demand for ramps and extended door frames,
- Hoists and lifting mechanisms (a device for vertical lifting of the disabled),
- Floors of different texture.

### 3.3. Mobility Limitations and the Demand for Assistance in Moving Outside the Apartment/House vs. The Respondents’ Age

Another problem analysed in the study was examining the relationship between the respondents’ age vs. his/her mobility limitations and the need for assistance while moving outside the apartment/house. The respective analyses were carried using chi-square test of independence or Fisher’s exact test.

**Figure 5:**
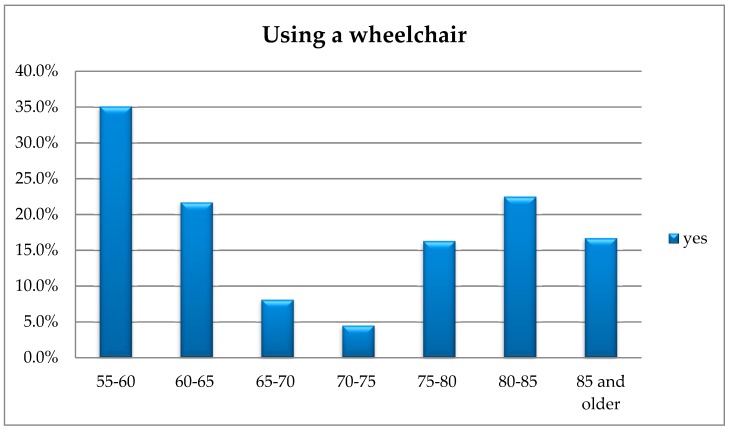
The respondents’ age vs. using a wheelchair.

The analysis using Fisher’s exact test showed significant differences regarding using wheelchairs in terms of the respondents’ age (*p* = 0.042). In order to check the nature of the existing differences, additional post hoc analyses were carried out, including the method by Baesley and Schumacker [29]. The analysis showed that people aged 55–60 use wheelchairs significantly more often than those aged 65–70 and 70–75. Figure 5 presents the responses provided by the respondents.

Such correlation is relatively difficult to explain, and rather means that using a wheelchair to move cannot be associated with the age of Lower Silesia voivodship residents.

The general overview of the discussed figure, however, allows formulating other conclusions, namely, wheelchairs are used by persons with disabilities who became disabled in earlier years and also people aged 75 and older. The increase in wheelchair users is observed in this particular age group.

The conducted research has shown that the number of people holding a disability certificate and using a wheelchair or a walking frame is significantly higher compared to those who do not have such a certificate. Indeed, there are more people in a wheelchair and using funds for the elimination of architectural and technical barriers than those who do not use such funds.

**Figure 6:**
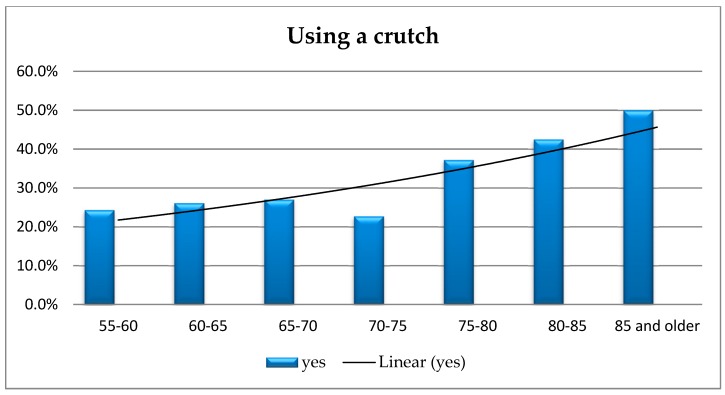
The respondents’ age vs. using a crutch.

The analysis using Fisher’s exact test did not show any significant differences regarding such observations as using a crutch when moving outside an apartment/house depending on the respondents’ age (*p* = 0.417). However, based on Figure 5, a conclusion can be drawn that, in the area of in Lower Silesia voivodship, people aged 85 and older use a crutch much more often—regardless of the disability certificate held (Figure 6). 

The amount of people using a crutch evidently increases at the age of 75 and continues in this tendency among the elderly. The research has not shown any significant correlation between the respondents’ age and using a wheelchair or a crutch outside their homes. In the latter case, an upward trend among people over 75 was observed (they most often use a crutch at the age of 85 and over regardless of the disability certificate). A study conducted in Germany, covering a large group of senior citizens (*n* = 4117) shows that approximately every second person suffers from a disability (44.7%). The problems concerned predominantly women, people with low incomes, and people suffering from joint or eye diseases [30].

**Figure 7:**
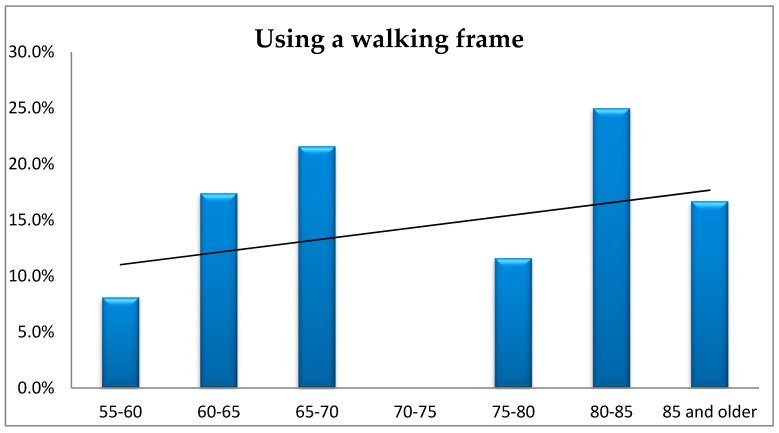
The respondents’ age vs. using a walking frame.

The findings did not show that the elderly used a walking frame more often, taking into account the respondents’ age (*p* = 0.075). Nevertheless, the largest proportion of such cases refers to people aged 80–85 (Figure 7).

**Figure 8:**
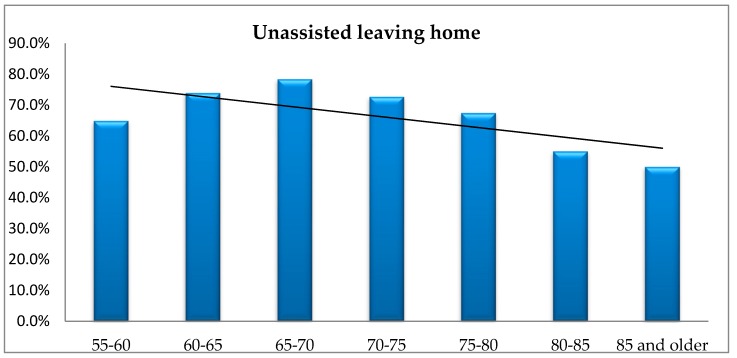
The respondents’ age vs. unassisted leaving home.

In order to check if the frequency of the unassisted leaving home is related to the respondents’ age, the respective analyses were carried out using Fisher’s exact test, which showed no significant differences between (*p* = 0.370) groups. Figure 8 illustrates the respondents’ answers.

The figure, however, indicates a downward trend in unassisted leaving home by the respondents from Lower Silesia voivodship, associated with their age. Starting from the age of 70, a decreasing tendency in the respondents’ unassisted leaving home can be observed.

**Figure 9:**
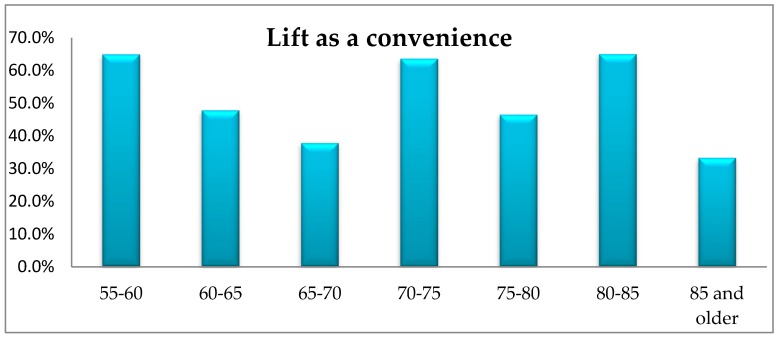
The respondents’ age vs. their opinion about the need of a lift in their immediate environment.

**Figure 10:**
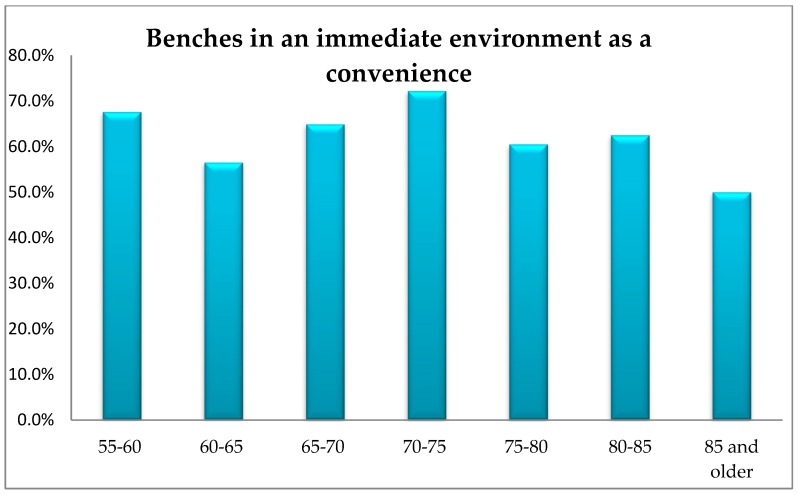
The respondents’ age vs. their opinion about the need of a bench in their immediate environment.

The conducted research did not show any significant differences in the respondents’ opinion depending on their age in terms of the demand for slipways, driveways, ramps, additional handrails along the walls, lifting equipment in the respondents’ immediate environment, lifts or benches. However, a significant proportion of the respondents identify the need to install a lift in the building or a bench in the close proximity of his/her apartment/house (Figure 9 and Figure 10).

The scale of demand for lifts and benches in all age groups was very high.

## 4. Services in The Respondents’ Environment

Having surveyed the demand for services in the immediate environment, it was established that all respondents, regardless of their age and place of residence, indicated the need for a health care centre in close proximity, they equally frequently pointed to the need of a grocery store (95% on average in each age group), a public transport stop (approx. 70% of the respondents) and a church (approx. 70% of respondents). These people need legible and familiar indoor and outdoor environments [31]. Such spaces are also beneficial to the community as a whole. Interesting examples come from The Netherlands and Belgium, where the Dementia-Friendly Municipalities initiative provides programmes for raising awareness among people about dementia and make services more accessible through training [32].

The analysis using Fisher’s exact test showed significant differences in the respondents’ indications regarding the proximity of a public transport stop related to their place of residence (*p* = 0.003). The respondents living in a city of over 250,000 residents indicate, significantly more frequently, the need of a bus stop in close proximity than those living in villages or in cities of up to 50,000 residents. It was also established that people living in villages more often express the need for a church in their immediate environment than people living in cities of up to 100,000, 250,000 and more than 250,000 residents.

**Figure 11:**
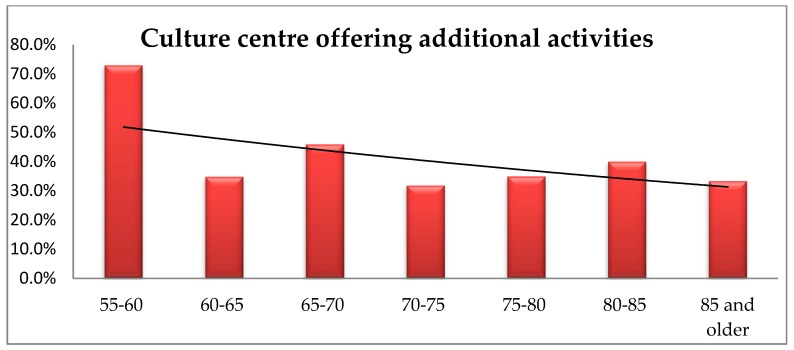
The respondents’ age vs. their opinion about the presence of a culture centre offering additional activities in their immediate environment.

The respondents representing younger age groups more often pointed to the demand for a community centre offering activities for senior citizens. The analysis using Fisher’s exact test showed significant differences in the respondents’ indications depending on the age group (*p* = 0.009). A post hoc analysis showed that people aged 55–60 significantly more often report such need than the remaining groups of respondents (Figure 11).

The need for such activities offer was reported by both the residents of villages and large cities. Many elderly persons would like to engage in out-of-home activities more often, but transportation deficiencies constitute a main obstacle [33,34,35].

**Figure 12:**
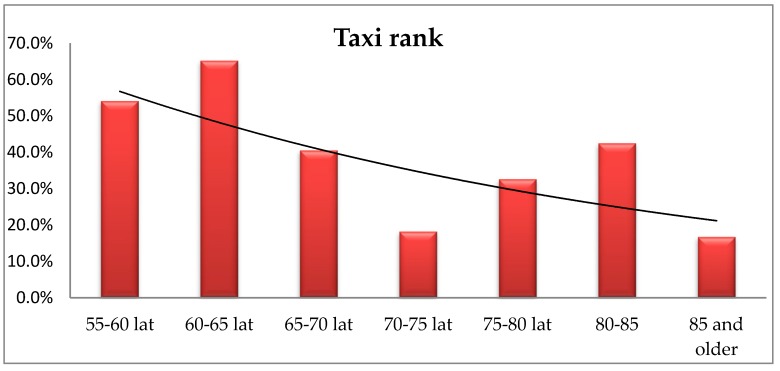
The respondents’ age vs. their opinion about the need of a taxi rank in their immediate environment.

Accessible public transport may help older persons to stay mobile longer in life and is important when visiting family and friends, for shopping and for physical exercise and sport events, among other activities [36,37]. The analysis using Fisher’s exact test showed significant differences in the provided responses regarding the need for a taxi rank in the immediate environment related to the respondents’ age (*p* = 0.015). A post hoc analysis showed that people aged 70–75 significantly less often indicate the need for access to a taxi rank than people aged 55–60 and 60–65. The results are illustrated on Figure 12.

The chi-square test of independence showed significant differences in the answers provided by the respondents’ regarding their place of residence (χ^2^(4) = 29,17; *p* < 0.001; *V* = 0.37). Those living in rural areas indicated significantly less often than other groups, the need for a taxi rank in their nearest environment. The respondents living in cities of up to 250,000 residents indicated such a need the most often and significantly more often than the respondents living in cities with over 250,000 residents, the cities of up to 100,000 residents and those living in villages.

**Figure 13:**
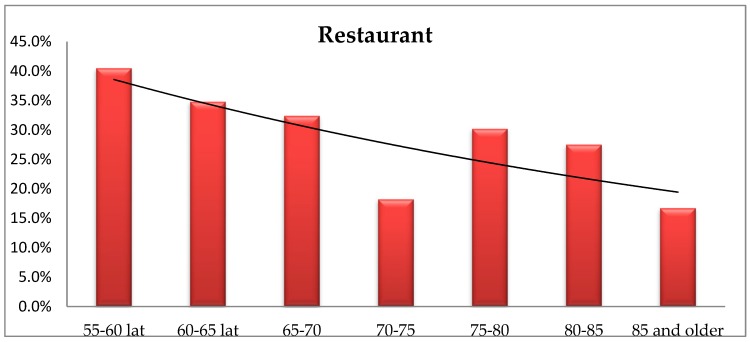
The respondents’ age vs. the opinion on the need of a restaurant in their immediate environment.

The demand for going out to a restaurant declines with age, although Fisher’s exact test did not show any significant differences in the frequency of provided responses by age (*p* = 0,675). The percentage of responses (Figure 13) indicates the occurring correlation of preferences depending on the respondent’s age.

The analysis using Fisher’s exact test showed significant differences in the provided responses regarding the place of residence (*p* = 0.026). The respondents living in cities of up to 250,000 residents indicate, significantly more often, the need of restaurants in their immediate environment comparing to those living in smaller towns and villages. People living in cities of over 250,000 population significantly more often point to the need of restaurants in their close proximity than the residents of cities up to 100,000 population as well as those living in villages.

## 5. Conclusions

It seems that, within the framework of social policy, more attention should be paid to the needs of senior citizens with disabilities, including those without the appropriate disability certificates, and thus the need to develop adequate support instruments. It seems like a well-grounded need to facilitate and extend access to the disability certification system. As it has already been mentioned, the activities aimed at the elimination of architectural barriers in the senior citizens’ place of residence and in their immediate environment are important in the context of improving their quality of life and independence. In other words, physical activity improves independence and well-being of the elderly. The conclusions resulting from the presented research, if implemented, may affect the everyday functioning of people suffering reduced mobility. Among senior citizens, there is a large group of the officially “fit” people (without appropriate certificates), however, suffering significant health limitations. These are, to a large extent, are the so-called “only biologically” disabled.

The respondents reported high demand for public utility services such as a health care centre, a grocery store, a public transport stop and a church. The elderly living in a larger city significantly more often indicated the need for a bus stop in their close proximity than those living rural areas, whereas younger people more frequently pointed to the need of a culture centre offering activities for senior citizens. The proximity of a taxi rank was most important for people living in cities of up to 250,000 population in the 55–65 age group. The demand for going out to a restaurant declines with age, however, it is most important is for the respondents living in cities of over 100,000 residents. Social exclusion arising from transport exclusion and the web of complex relationships between exclusion of various types is, then, a major challenge to wellbeing as people move through the course of life, when disability, financial barriers or lack of accessibility potentially restrict transport availability and choice [38,39]. 

The presented conclusions make it clear that the adopted research Hypothesis 2 was confirmed. Hypothese 1 was not confirmed in the results of younger groups and primarily refers to elderly people aged 75 and older. No significant differences were recorded between the particular age groups of the respondents regarding the frequency of leaving their apartments/houses unassisted, however, a declining trend in this phenomenon was observed along with age. All age groups were characterised by a high scale of demand for lifts in residential buildings and benches in the immediate environment.

Based on the conducted research, recommendations for policy-makers and local authorities were formulated, the implementation of which may contribute to the improvement of senior citizens’ living comfort:

1. A system of facilities for senior citizens should be provided allowing access to the physicians issuing disability certificates. At present the disability degree is decided by county/municipal disability evaluation boards—as the first instance and voivodship teams—as the second instance. However, the mobility of seniors is limited and without the help of another person, accessing the county centre where the disability evaluation board is seated remains practically impossible. In turn, the individuals who do not hold the disability certificate are not entitled to receive funding for the elimination of architectural barriers.
2. The demographic situation of the country will force, in the future, the changed perception of the problem faced by the elderly and the current support system for the disabled. Along with the increase in the number of post-productive age population, the entire system change will become an essential element in improving the living comfort of older people. There has been a recent emphasis on “ageing-in-place” in research and policy. This is defined as “remaining living in the community, with some level of independence, rather than in residential care” [40].
3. Ultimately, it is crucial to develop a funding system, operating independently from the system addressed to people with disabilities (currently PEFRON—The State Fund for Rehabilitation of Disabled People), which allows obtaining financial means by the elderly to eliminate architectural barriers in their environment (apartment/house). One of the criteria for awarding financial resources would be the person’s age. The system could be financially supported from the state budget. In such a situation, legal regulations and earmarked funds should be developed to help senior citizens.
4. The new system aimed at supporting seniors should operate independently from the current system focused on helping people with disabilities at the municipality level (the smallest unit of the country administrative division). The respective assistance should be organized comprehensively, including delegating a special renovation team to carry out renovation works in the senior citizens’ apartments/houses. There is also broad agreement that helping people to continue to live in their own homes is desirable on economic grounds as it is less expensive than options such as residential care. Physical modifications to the original structure and design of dwellings, especially in relation to improving accessibility of home environments, have also been shown to be key elements in facilitating aging-in-place [41,42].

## Figures and Tables

**Table 1:** Mobility limitations vs. the disability certificates held.

Mobility Limitations Resulting in the Need to Use the Following While Moving	Answer	Are You A Disability Certificate Holder?				χ^2^	*p*	φ
		No		Yes				
		*n*	%	*n*	%			
Wheelchair	no	107	62.6	8	20.0	23.69	<0.001	0.34
	yes	64	37.4	32	80.0			
Crutch	no	81	55.9	34	51.5			
	yes	64	44.1	32	48.5	0.35	0.557	0.04
Walking frame	no	106	59.2	9	28.1			
	yes	73	40.8	23	71.9	10.58	0.001	0.22

*Note: n*—number; %—percent; χ^2^—chi-square test of independence value; *p*—significance level; φ—effect size.

**Table 2:** Mobility limitations vs. using state funds for the elimination of architectural and technical barriers.

Mobility Limitations Resulting in the Need to Use the Following While Moving	Answer	Have You Used State Funds for the Elimination of Architectural And Technical Barriers?				χ^2^	*p*	φ
		No		Yes				
		*n*	%	*n*	%			
Wheelchair	no	158	92.4	32	80.0		0.035 ^a^	
	yes	13	7.6	8	20.0			
Crutch	no	131	90.3	59	89.4	0.05	0.831	0.02
	yes	14	9.7	7	10.6			
Walking frame	no	163	91.1	27	84.4		0.331 ^a^	
	yes	16	8.9	5	15.6			

*Note: n*—number; %—percent; χ^2^—chi-square test of independence value; *p*—significance level; φ—effect size; ^a^—Fisher’s exact test analysis.

## Supplementary Materials

The following are available online at https://www.mdpi.com/1660-4601/16/14/2601/s1, Supplementary Material 1: Questions asked in the survey questionnaire.

## References

[1] Van Hoof J., Kazak J. (2018). Urban ageing. Indoor Built Environ..

[B2-ijerph-16-02601] 2.The Act of 27 August 1997 on Vocational and Social Rehabilitation and Employment of Persons with Disabilities (Journal of Laws of 2011, No. 127 item 721 consolidated text) (Journal of Laws from 2011 No. 127 item 721 uniform text).

[3] Henderson V. (2002). Urban primacy, external costs, and quality of life. Resour. Energy Econ..

[4] Przybyła K., Kulczyk-Dynowska A., Kachniarz M. (2014). Quality of Life in the Regional Capitals of Poland. J. Econ. Issues.

[5] Colombo E., Michelangeli A., Stanca L. (2014). *La Dolce Vita*: Hedonic Estimates of Quality of Life in Italian Cities. Reg. Stud..

[6] Twardzik M., Wrana K., Klasik A., Kuźnik F. (2017). Podstawy i kierunki kształtowania witalności miast [Basics and directions for developing city vitality]. Nowe Praktyki Rozwoju Lokalnego i Regionalnego [New Practices Inlocal and Regional Development].

[7] Bartkowski J. (2012). Położenie społeczne i ekonomiczne zbiorowości osób niepełnosprawnych w Polsce na podstawie demograficznych danych zastanych i ustaleń badań przeprowadzonych w ostatnich pięciu latach.

[8] Frąckiewicz L. (2001). Demograficzno-społeczne problemy osób niepełnosprawnych [Demographic and social problems faced by persons with disabilities]. Polityka Społeczna.

[9] Piatkowski M. (2018). Europe’s Growth Champion. Insights from the Economic Rise of Poland.

[10] Przybyła K., Kulczyk-Dynowska A. (2018). Transformations and the Level of Tourist Function Development in Polish Voivodeship Capital Cities. Sustainability.

[11] Gasińska M. (2016). Dochody gospodarstw domowych w Polsce—Wybrane obiektywne i subiektywne ujęcia i dane [Incomes of Households in Poland—the Selected Objective and Subjective Approaches and Data]. Zeszyty Naukowe Uczelni Vistula.

[12] Babczuk A., Kachniarz M. (2012). Sources of soft budget constraints in the structure of autonomous public healthcare centres. Argum. Oecon..

[13] Hełdak M., Stacherzak A., Przybyła K. (2018). Demand and Financial Constraints in Eliminating Architectural and Technical Barriers for People with Disabilities in Poland. J. Healthc. Eng..

[14] Gąciarz B., Bartkowski J. (2012). Położenie społeczno-ekonomiczne niepełnosprawnych w Polsce na tle sytuacji osób niepełnosprawnych w krajach Unii Europejskiej [Social and economic situation of persons with disabilities in Poland compared to the situation of the disabled in the European Union Member States]. Niepełnosprawność Zagadnienia Problemy Rozwiązania [Disability Issues Prob. Solutions].

[15] Raszkowski A., Bartniczak B. (2018). Towards Sustainable Regional Development. Economy, Society, Environment, Good Governance Based on the Example of Polish Regions. Transform. Bus. Econ..

[16] Raszkowski A., Głuszczuk D., Löster T., Pavelka T. (2017). Contemporary developmental directions of revitalization projects: Polish experiences. The 11th International Days of Statistics and Economics.

[17] Kazak J., van Hoof J., Świąder M., Szewrański S. (2018). Real estate for the ageing society—The perspective of a new market. Real Estate Manag. Valuat..

[18] Ciok S. (1995). Zmiany Ludnościowe i Osadnicze w Sudetach [Population and Settlement Changes in the Sudeten].

[19] Cieślak M. (1999). Procesy Demograficzne w Byłych Województwach Dolnośląskich w Latach 1945–1997 [Demographic Processes in Former Voivodships of Lower Silesia in the Period 1945–1997].

[20] Latocha A. (2012). Changes in the rural landscape of the Polish Sudety Mountains in the post-war period. Geogr. Pol..

[21] Górecka S., Szmytkie R. (2015). Prognoza Demograficzna dla Gmin Województwa Dolnośląskiego do 2035 Roku [Demographic Forecast for the Municipalities of Lower Silesia Voivodship Till 2035].

[22] Szmytkie R. (2016). Depopulacja zespołów miejskich w sudeckiej części Dolnego Śląska [Depopulation of urban centres in the Sudeten part of Lower Silesia]. Konserwatorium Wiedzy o Mieście.

[23] Stacherzak A., Hełdak M. (2019). Borough Development Dependent on Agricultural, Tourism, and Economy Levels. Sustainability.

[24] Central Statistical Office (2016). Information of the Minister of Health on the impact of demographic changes and population aging on the organization of the health care system and the National Health Program. The Sejm’s Senior Policy Commission Regarding.

[25] Wojtkowiak-Jakacka M., Girul A., Hrynkiewicz J., Potrykowska A. (2017). Sytuacja demograficzna województwa dolnośląskiego—stan obecny i perspektywy [Demographic situation of the Lower Silesia Voivodship-current status and prospects]. Sytuacja demograficzna Dolnego Śląska jako wyzwanie dla polityki społecznej i gospodarczej [Demographic situation of Lower Silesia as a challenge for social and economic policy].

[26] Central Statistical Office (2018). Demographic Yearbook.

[27] Kopeć B. (1983). Students Textbook of University of Agriculture in Wrocław No. 269. Metodyka badań ekonomicznych w gospodarstwach rolnych [Methodology of Economic Research in Agricultural Holdings].

[28] Stachak S. (1978). Students Textbook of University of Agriculture in Szczecin. Metody nauk ekonomiczno—Rolniczych w zarysie [The outline of methods in economic and agricultural sciences].

[29] Beasley T.M., Schumacker R.E. (1995). Multiple Regression Approach to Analyzing Contingency Tables: Post Hoc and Planned Comparison Procedures. J. Exp. Educ..

[30] Strobl R., Müller M., Emeny R., Peters A., Grill E. (2013). Distribution and determinants of functioning and disability in aged adults—Results from the German KORA-Age study. BMC Public Health.

[31] Van Hoof J., Kort H., Van Waarde H., Blom M. (2010). Environmental Interventions and the Design of Homes for Older Adults with Dementia: An Overview. Am. J. Alzheimer’s Dis. Other Dement..

[32] Van Hoof J., Kazak J.K., Perek-Białas J.M., Peek S.T.M. (2018). The Challenges of Urban Ageing: Making Cities Age-Friendly in Europe. Int. J. Environ. Res. Public Health.

[33] Gabriel Z., Bowling A. (2004). Quality of life from the perspectives of older people. Ageing Soc..

[34] Farquhar M. (1995). Elderly people’s definitions of quality of life. Soc. Sci. Med..

[35] Su F., Bell M.G. (2009). Transport for older people: Characteristics and solutions. Res. Transp. Econ..

[36] Sundling C., Berglund B., Nilsson M.E., Emardson R., Pendrill L.R. (2014). Overall Accessibility to Traveling by Rail for the Elderly with and without Functional Limitations: The Whole-Trip Perspective. Int. J. Environ. Res. Public Health.

[37] Linder P. (2007). Äldre människors res-och aktivitetsmönster—En litteraturstudie. Older Persons’ Travel and Activity Patterns.

[38] Church A., Frost M., Sullivan K. (2000). Transport and social exclusion in London. Transp. Policy.

[39] Rye T., Mykura W. (2009). Concessionary bus fares for older people in Scotland—Are they achieving their objectives?. J. Transp. Geogr..

[40] Davey J., de Joux V., Nana G., Arcus M. (2004). Accommodation Options for Older People in Aotearoa/New Zealand. https://www.beehive.govt.nz/sites/all/files/Accomodation%20Options%20for%20Older%20People.pdf.

[41] Hwang E., Cummings L., Sixsmith A., Sixsmith J. (2011). Impacts of Home Modifications on Aging-in-Place. J. Hous. Elder..

[42] Fox S., Kenny L., Day M.R., O’Connell C., Finnerty J., Timmons S. (2017). Exploring the Housing Needs of Older People in Standard and Sheltered Social Housing. Gerontol Geriatr Med..

