# Supplementary material for: The Actual Demand for the Elimination of Architectural Barriers among Senior Citizens in Poland
Source: Int J Environ Res Public Health. 2019 Jul 22;16(14):2601. doi: 10.3390/ijerph16142601 (PMC6678534; doi:10.3390/ijerph16142601)
Supplement: Supplementary file 1 [file ijerph-16-02601-s001.pdf]

Annex 1. Questions asked in the survey questionnaire.

<u>Research on the actual number of people reporting the need to eliminate architectural and technical barriers in their environment</u>

1. ***Have you used state funds granted for the elimination of architectural and technical barriers:***

- *Yes*
- *No*

2. ***Do you have facilities adapting your apartment/house to your needs:***

o *Various handholds / grippers*	o *Extended door frames*
o *Adjusted bathrooms / toilets*	o *Hoists and lifting mechanisms*
o *Slip resistant flooring*	o *Different texture / colour floors*
o *Additional handrails along the walls*	o *Lifting equipment*
o *Adjusted thresholds for doors*	o *None of the above*
o *Ramps*	o *Other, what kind ……………………………..*

3. ***Which of the following facilities should be adapted to adjust your apartment/house to your needs and facilitate moving around:***

o *Various handholds / grippers*	o *Extended door frames*
o *Adjusted bathrooms / toilets*	o *Hoists and lifting mechanisms*
o *Slip resistant flooring*	o *Different texture / colour floors*
o *Additional handrails along the walls*	o *Lifting equipment*
o *Adjusted thresholds for doors*	o *None of the above*
o *Ramps*	o *Other, what kind ……………………………..*

4. ***Dou you have a disability certificate:***

- *Yes*
- *No*

5. ***If yes, the certificate refers to:***

- *significant disability*
- *moderate disability*
- *slight disability*

6. ***Do you have a mobility limitation requiring to use the following while moving:***

- *wheelchair;*
- *crutch;*
- *walking frame*
- *other, what kind ……………………………………………………………………………………………*

7. ***Do you leave your apartment/house on your own:***

- *Yes*
- *No*

8. ***If not, who helps you in moving around:***

- *family member*
- *social worker*
- *other people, who ………………………………………………………*

9. Please indicate what mobility facilities you need to move in your immediate environment (neighbourhood) - outside your apartment/house:	
o *Slipways*	o *Additional handrails along the walls*
o *Driveways*	o *Extended door frames*
o *Ramps*	o *Different texture / colour pavements*
o *Lift*	o *Lifting equipment*
o *Benches in close proximity*	o *None of the above*
o *Various handholds / grippers*	o *Other, what kind ………………………………*
10. The respondent's place of residence:	
o *Detached house*	
o *Terraced house*	
o *Apartment in a multi-family building (block of flats)*	
o *Nursing home*	
o *Other, what kind …………………………………………………………………….*	
11. The respondent's family situation:	
o *I live alone*	
o *I live with my husband/wife*	
o *I live with my husband/wife and children*	
o *I live with my children*	
o *I live with (my parents, sister, brother, extended family)*delete as appropriate*	
o *Nursing home*	
o *Other, what kind …………………………………………………………………….*	
12. Age range:	
o *55-60*	
o *60-65*	
o *65-70*	
o *70-75*	
o *75-80*	
o *80-85*	
o *85 and older*	
13. Place of residence:	
o *village*	
o *city up to 50 000 residents*	
o *city up to 100 000 residents*	
o *city up to 250 000 residents*	
o *city over 250 000 residents*	
14. Gender:	
o *female*	
o *male*	

Source: Authors' compilation
